# Use of the OSCE in assessing performance in first aid of high school students

**DOI:** 10.31744/einstein_journal/2026AO1865

**Published:** 2026-04-22

**Authors:** Vinicyus Eduardo Melo Amorim, Renan Gatis Ayres, Mikhael Morais de Souza, Ana Cecília Araújo Cabral, Bruna Sampaio Tavares, Tiago Fernando Ferreira da Silva, Felipe César Gomes de Andrade, Luciano Calheiros de Morais Guerra, Edvaldo da Silva Souza

**Affiliations:** 1 Faculdade Pernambucana de Saúde Recife PE Brazil Faculdade Pernambucana de Saúde, Recife, PE, Brazil.

**Keywords:** First aid, Cardiopulmonary resuscitation, Gagging, Competency-based education, Education, primary and secondary

## Abstract

**Objective:**

To assess the knowledge and performance of high school students in cardiopulmonary resuscitation and foreign body airway obstruction management using an objective structured clinical examination.

**Methods:**

An interventional design was implemented with high school students, who were assessed before and after the intervention. Participants completed a theoretical questionnaire and two objective structured clinical examinations—one for cardiopulmonary resuscitation and one for foreign body airway obstruction—before receiving theoretical and practical first aid training. Subsequent assessments included the same theoretical questionnaire and objective structured clinical examinations, administered immediately after training and again after six months to measure knowledge retention.

**Results:**

Comparison of pre-training, immediate post-training, and six-month follow-up data revealed significant improvements in knowledge and performance across all student groups and scenarios (p<0.001), with sustained retention over time. Implications for School Health Policy, Practice, and Equity: Utilizing handmade mannequins alongside the objective structured clinical examination methodology offers a cost-effective approach for training students and mitigating adverse outcomes in high schools.

**Conclusion:**

The objective structured clinical examination is an effective and evaluative tool for first aid training among high school students.

## HIGHLIGHTS

■ Training markedly improves theoretical knowledge and performance in cardiopulmonary resuscitation and foreign body airway obstruction management.

■ Significant knowledge and skills, particularly in the Heimlich maneuver, remain after 6 months.

■ The objective structured clinical examination is a practical and reliable method to evaluate first-aid skills in schools.

## In Brief

High school students who underwent both classroom and handson first aid training showed notable gains in their knowledge and performance of cardiopulmonary resuscitation and the Heimlich maneuver. After six months, students retained a substantial portion of their skills, demonstrating the effectiveness and feasibility of using the objective structured clinical examination method as a teaching and assessment tool.

## Graphical abstract

**Figure:**
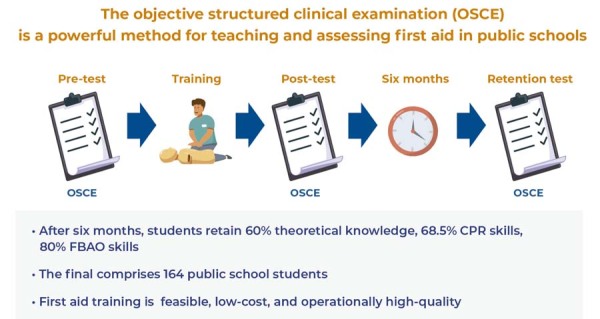

## INTRODUCTION

First aid comprises a series of immediate interventions administered to individuals experiencing life-threatening emergencies.^(1)^ Cardiopulmonary resuscitation (CPR) performed by bystanders improves survival rates and decreases the risk of long-term sequelae.^(2-6)^ Accordingly, training laypeople is essential, as those with proper instruction are more capable of delivering effective first aid and are more likely to respond during emergencies.^(7)^

Historically, it was assumed that only adults could provide adequate assistance in emergency scenarios. However, recent research has established that children aged 5 to 18, when appropriately trained, can identify risk scenarios and recognize vital signs in critically ill individuals.^(8-12)^ Hence, expanding basic life support (BLS) training to students could potentially reduce mortality rates, given the demonstrated willingness of many young people to offer assistance.^(7,13)^

The high rates of morbidity and mortality among children and adolescents due to external causes have underscored the necessity of first-aid education within schools. The effectiveness of active learning and objective structured clinical examination (OSCE) methods in health-related fields has been demonstrated by evaluating professionals’ competencies and skills within specific timeframes. Such approaches enhance the psychometric rigor of assessments and foster continuous improvements in learning standards across diverse clinical settings.^(14-16)^

Nevertheless, integrating first-aid education in schools, particularly at the high school level, presents notable challenges. The high cost of simulation mannequins, limited access to suitable logistical environments, and the absence of a well-established culture of practical training impede the adoption of assessment tools, such as the OSCE, among this demographic. Moreover, OSCE usage in Brazil remains relatively nascent, having been introduced in 1991 at the State University of Londrina, supported by international partnerships with McMaster University and Maastricht University. Cumulatively, these factors complicate the implementation of such initiatives in high school contexts, contributing to the scarcity of related studies and publications.

## OBJECTIVE

This study assess high school students’ knowledge and performance in cardiopulmonary resuscitation and foreign body airway obstruction scenarios using the objective structured clinical examination method.

## METHODS

### Design

A quasi-experimental, pre-post intervention design without a control group was utilized to assess the effectiveness of a structured educational intervention on the acquisition of BLS competencies among high school students.

### Setting

The study was conducted at a Brazilian medical school and included students in the early years of their undergraduate studies. Educational sessions were provided in classroom environments, using low-cost, handcrafted manikins to facilitate hands-on BLS training. This approach underscores the significance of cost-efficient clinical skills education for public institutions, particularly in resource-limited contexts typical of low- and middle-income countries.

### Participants

The target population comprised students officially enrolled in any high school class at the *Escola de Referência em Ensino Médio Fernando Mota* (located in Recife) and without medical restrictions that would preclude participation in physical activities. The school comprises 15 high school classes, totalling 600 students. Participants were recruited through non-probabilistic convenience sampling.

**Figure 1:**
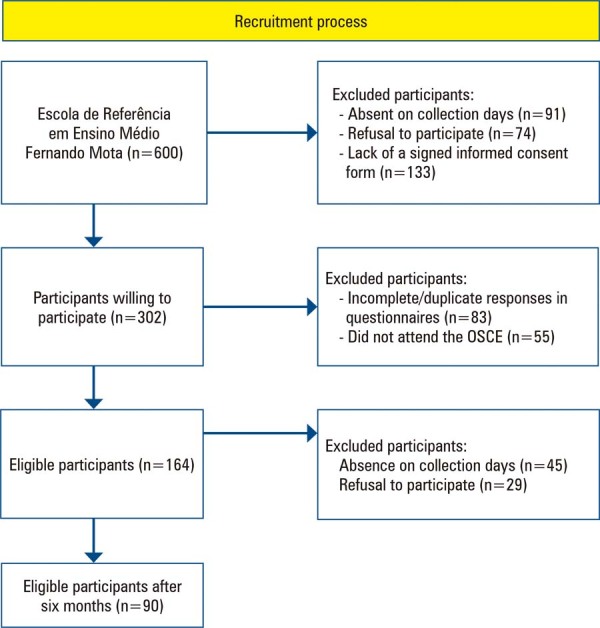
Student recruitment flowchart

Researchers visited all classrooms in person to identify students interested in participating in the project. Each stage of the participation process was clearly explained during these visits. As an incentive, volunteers who completed all phases of the study were offered a chocolate bar. Despite the large number of students enrolled at the institution, not all were present in their respective classrooms on the day the survey was conducted. Additionally, students who declined to participate or did not provide a signed consent form from their legal guardians were excluded from the study. Figure 1 presents a flowchart depicting participant recruitment and adherence**.**

### Data collection and measurements

#### Instruments

Four data collection instruments were used at different stages of the study. A theoretical questionnaire (TQ) comprised two sections: a sociodemographic assessment (including sex, age, students’ and parents’ years of education, school failure rates, and number of previous first-aid training sessions), and a specific evaluation of CPR and foreign body airway obstruction (FBAO) knowledge.

The specific assessment comprised ten multiple-choice questions, each with four possible responses, designed to evaluate baseline knowledge of relevant scenarios. Each item was assigned 1 point, for a maximum score of 10. Question parameters were developed according to the American Heart Association (AHA) guidelines.^(17)^

The second and third instruments were the OSCEs, intended to assess students’ competencies in two simulation stations developed by the researchers: a cardiopulmonary arrest scenario and an FBAO scenario.

The CPR OSCE incorporated eight parameters, each worth one point, based on the AHA guidelines: evaluating victim responsiveness, checking the carotid pulse and respiration, initiating effective CPR (with proper hand positioning, compression rate of 100-120/min, compression depth of 5-6 cm, and full chest recoil), requesting emergency medical services and an automated external defibrillator (AED), correct AED placement, stepping back during defibrillation, reassessing the carotid pulse for spontaneous circulation return, and resuming CPR after the first cycle. If a student did not request an AED, points for stepping back and AED placement were automatically not awarded. To better reflect the context where AEDs are often unavailable in Brazil, performance was also evaluated excluding these two criteria, resulting in an adjusted maximum score of 6.0 points.

Cardiopulmonary resuscitation simulation mannequins were handcrafted by the researchers using recycled clothing for the outer layer and polyethylene terephthalate bottles to simulate compression springs. The only commercially acquired device was an AED for simulation.

The FBAO OSCE comprised three parameters, each assigned one point: initial communication with the choking victim; initiating the Heimlich maneuver; and effectively performing the Heimlich maneuver (including correct hand positioning, the appropriate compression site, and execution of the “J” motion). Researchers acted as simulated choking victims.

The fourth instrument, administered at the end of the study, was a satisfaction survey containing ten items rated on a 5-point Likert scale (1 = strongly disagree, 5 = strongly agree). The survey evaluated participants’ likelihood to recommend the program, satisfaction with the program, perceptions of the organization and duration, difficulty of the theoretical questionnaire, self-confidence in responding to CPR and FBAO scenarios, satisfaction with feedback, and perceived learning and progress throughout the training program.

#### Procedure

This study adopted an interventional, uncontrolled, pre- and post-assessment design, conducted in four distinct phases: pilot, pre-test, immediate post-test, and final assessment. The pilot phase involved a reduced sample of 21 students from the institution, who were subsequently excluded from the final analysis. The purpose of this phase was to train the research team and identify potential operational issues.

The research team comprised medical students who had received prior training from faculty physicians at Faculdade Pernambucana de Saúde. These student researchers actively participated in the evaluation and simulation scenarios, including role-playing with standardized patients. A total of ten sessions were required to evaluate all student groups, with each session lasting approximately seven hours: four hours in the morning—two for preliminary assessments and two for training—and three hours in the afternoon dedicated to post-intervention questionnaires and OSCEs.

During the pre-test phase, participants completed a TQ containing sociodemographic items and scenario-specific evaluative questions. This was followed by two OSCEs (CPR and FBAO) to assess baseline performance before training. The subsequent training phase, lasting two hours, included theoretical and practical instruction on the scenarios. Key objectives encompassed understanding cardiopulmonary arrest concepts and recognition parameters; differentiating between cardiopulmonary arrest and fainting; identifying emergency service numbers and appropriate timing for calls; patient positioning during cardiopulmonary arrest; mastering effective CPR techniques; learning AED concepts and applications; recognizing partial or total airway obstruction; and performing the Heimlich maneuver in adults, children, newborns, and pregnant women.

After training, the immediate post-test phase was conducted, during which students completed a second theoretical questionnaire identical to the initial one and repeated the same two OSCEs, allowing for performance comparison post-training. During this period, students did not have access to the answer keys. Upon completing the OSCEs, individualized feedback was provided, highlighting strengths and areas requiring improvement. Students also completed a satisfaction survey to assess the training program.

The final phase occurred six months post-training, evaluating long-term knowledge retention and performance via readministration of the theoretical questionnaire and the same CPR and FBAO OSCEs, without additional training.

Students were allotted up to five minutes per simulation scenario. However, most used only half of the available time, either because they completed the scenario or, more commonly in the pre-training phase, because they terminated early due to uncertainty about the appropriate steps.

## Data analysis

Student knowledge and performance were analyzed using Jamovi version 2.3.21 for Windows and Excel 2016. Statistical tests were performed with a 95% confidence interval, and results with p-values <0.05 were considered statistically significant. Measures of central tendency and dispersion were used to describe numerical variables. The Shapiro-Wilk test assessed normality, while the Wilcoxon, Mann-Whitney, and Kruskal-Wallis tests were applied to pre- and post-analyses. Pearson’s Correlation Test (PCT) was used to evaluate correlations.

To determine the percentage of knowledge and skill retention six months after training, the following formula was applied:


 Retention Percentage =[1-(knowledge loss after six months)/(knowledge gain after training)]×100

where *Knowledge loss over six months* = mean score immediately after training - Mean score six months later; *Knowledge gain after training* = mean score immediately after training - mean score before training.

Additionally, mean rankings were used to interpret Likert scale scores. Reliability of the satisfaction questionnaire was assessed using Cronbach’s alpha, with values greater than 0.7 indicating acceptable reliability for each variable.

## Ethical considerations

This study was approved by the Ethics Committee of *Faculdade Pernambucana de Saúde* under CAAE: 58804122.0.0000.5569; #5.433.839.

## RESULTS

Following verification of eligibility criteria, 302 students were included in the study (Figure 1). Six months after training, 90 students returned to participate in the fourth phase, which assessed knowledge retention and longitudinal performance. Despite sample loss in the final analysis, the epidemiological profiles of participants remained statistically consistent across all parameters (p>0.05; Table 1).

**Table 1:** Descriptive analysis of students’ sociodemographic characteristics

	Immediate post-training phase (n=164)	Six months post-training phase (n=90)	p value
Average age	16.1	16.2	0.592
Sex - n (%)			
Male	66 (40.2)	39 (43.3)	0.632
Female	98 (59.8)	51 (56.7)	
Series - n (%)			
1st year	40 (24.4)	23 (25.6)	0.369
2nd year	70 (42.6)	44 (48.8)	
3rd year	54 (33)	54 (33)	
Carrying out prior training - n (%)			
Yes	30 (18.3)	14 (15.6)	0.567
No	134 (81.7)	76 (84.6)	
Maternal education - n (%)			
Illiterate	1 (0.6)	1 (1.1)	
IEE	11 (6.7)	5 (5.5)	0.781
CEE	13 (7.9)	8 (8.9)	
High school	72 (43.9)	38 (42.2)	
Higher education	46 (28)	24 (26.7)	
Unable to provide information	21 (12.8)	14 (15.5)	
Paternal education - n (%)			
Illiterate	2 (1.2)	0 (0)	0.549
IEE	13 (7.9)	6 (6.7)	
CEE	14 (8.5)	6 (6.7)	
High school	62 (37.8)	35 (38.3)	
Higher education	31 (18.9)	20 (22.2)	
Unable to provide information	42 (25.6)	19 (21.1)	
School failures - n (%)			
Yes	14 (8.5)	10 (11.1)	0.502
No	150 (91.5)	80 (88.9)	

IEE: Incomplete elementary education; CEE: complete elementary education.

Post-training analyses indicated a statistically significant improvement in knowledge and performance (Table 2). Comparisons of student outcomes across the first, second, and third questionnaire administrations revealed significant differences (p<0.001) in all analyses, except the comparison between the second and third FBAO OSCEs (p=0.02). The analysis of CPR OSCE scores, exclusive of DEA parameters, remained significant (p<0.001).

**Table 2:** Scores obtained in the theoretical questionnaires and cardiopulmonary resuscitation and foreign body airway obstruction objective structured clinical examination

Evaluation method	Maximum possible score	1^st^ IQR	Md	3^rd^ IQR
1^st^ Theoretical questionnaire	10.00	3.00	4.00	5.00
2^nd^ Theoretical questionnaire	10.00	7.00	8.00	9.00
3^rd^ Theoretical questionnaire	10.00	5.00	6.00	7.00
1^st^ CPR-OSCE	8.00	0.00	0.00	1.00
2^nd^ CPR-OSCE	8.00	2.00	4.50	7.00
3^rd^ CPR-OSCE	8.00	2.00	3.00	5.00
1^st^ CPR-OSCE without AED parameters	6.00	0.00	0.00	1.00
2^nd^ CPR-OSCE without AED parameters	6.00	2.00	4.00	5.00
3rd CPR-OSCE without AED parameters	6.00	0.00	0.00	3.00
1^st^ FBAO-OSCE	3.00	0.00	1.00	1.00
2^nd^ FBAO-OSCE	3.00	2.00	2.00	3.00
3^rd^ FBAO-OSCE	3.00	1.00	2.00	3.00

Md: median; IQR: interquartile range; OSCE: objective structured clinical examination; CPR: cardiopulmonary resuscitation; FBAO: foreign body airway obstruction.

Although questionnaire and OSCE scores declined six months post-training (Table 2), retention of knowledge, competencies, and skills remained significantly higher than baseline values (p<0.001).

At six months post-training, the median questionnaire score remained 50% higher than the baseline value, while the median CPR OSCE score decreased by approximately 33% compared with immediate post-training. No changes were observed in median FBAO OSCE scores (Table 3).

**Table 3:** Proportion of correct questionnaire and objective structured clinical examination answers before and after training by competence and skill

Skills assessed by question in theoretical questionnaires				
Competence assessed	% Correct answers in 1st TQ	% Correct answers in 2nd TQ	% Correct answers in 3rd TQ	pvalue
Emergency number	67.1	100	88.9	<0.001
Suitable pulse for CPR check	56.1	89.6	77.8	<0.001
Appropriate compression site for CPR	11.6	55.5	38.9	<0.001
Compression rate in CPR	9.1	75.0	31.1	<0.001
CPR parameters	14.6	50.6	28.9	<0.001
Description of the Heimlich maneuver	85.4	93.3	94.4	<0.05
Environmental safety chain in CPR scenarios	18.9	47.0	41.1	<0.001
Airway clearance maneuver in infants	80.5	87.2	92.2	0.07
Correct positioning of AED	6.7	92.7	63.3	<0.001
CPR diagnostic parameters	43.3	75.0	62.2	<0.001
Skills assessed in the CPR OSCE				
**Skill assessed**	**% correct answers in the 1st OSCE**	**% correct answers in the 2nd OSCE**	**% correct answers in the 3rd OSCE**	**p value**
Check if the patient responds	10.4	70.1	48,9	<0.001
Check carotid pulse and breathing	1.8	56.7	44,4	<0.001
Effective initiation of CPR	20.1	59.8	42,2	<0.001
Call emergency services and request the AED	4.3	41.5	44,4	<0.001
Correct positioning of AED	0.6	58.5	46,7	<0.001
Step away to defibrillate	0.6	40.2	10,0	<0.001
CPR restart	4.9	54.9	48,9	<0.001
Assess spontaneous circulation	8.5	59.1	34,4	<0.001
Skills assessed in the FBAO OSCE				
**Skill assessed**	**% correct answers in the 1st OSCE**	**% correct answers in the 2nd OSCE**	**% correct answers in the 3rd OSCE**	**p value**
Communication with victim	4.3	56.1	40.4	<0.001
Starting Heimlich maneuver	67.7	98.2	96.6	<0.001
Heimlich maneuver performed effectively	32.3	72.6	61.4	<0.001

All variables have a Shapiro-Wilks p>0.05; Wilcoxon test compared variables.

Md: median; TQ: theoretical questionnaire; OSCE: objective structured clinical examination; CPR: cardiopulmonary resuscitation; FBAO: foreign body airway obstruction; AED: automated external defibrillator.

An analysis of correlations between age and assessment results before and after training using PCT showed a weak negative association with questionnaire performance at six months post-training (p=0.014, Pearson’s R = -0.257). No other significant associations were observed. Regarding sex, only the post-training CPR OSCE score showed a statistically significant association (p=0.04), with female participants performing better.

Performance by grade level (1^st^, 2^nd^, and 3^rd^) showed no significant differences, as indicated by the Kruskal-Wallis test (p>0.05). Similarly, a history of academic failure and maternal education did not impact students’ knowledge or performance (p>0.05). However, paternal education was associated with CPR OSCE performance at six months post-training (p=0.013), with no other associations identified.

Comparing the group of students with prior first-aid training (18.29%) with those without, the only statistically significant difference in performance was observed in the pre-training FBAO OSCE (p<0.001). Prior training emerged as a positive determinant, with mean scores of 1.18 and 0.7, respectively.

At six months post-training, retention rates were as follows: 60% for theoretical knowledge, 68.5% for CPR OSCE-acquired skills, and 80% for FBAO OSCE-acquired skills. The pre-test questionnaire positively correlated with the pre-test CPR OSCE (p<0.05, PCT: very weak), but not with the pre-test FBAO OSCE. Post-test questionnaires were positively correlated with post-test CPR and FBAO OSCEs (p<0.001, PCT: moderate). After six months, the TQ maintained correlation only with the CPR OSCE (p=0.04, PCT: weak). Additionally, a positive correlation was observed between OSCEs immediately post-training (p<0.001, PCT: moderate) and six months post-training (p=0.017, PCT: weak). Positive correlations were also observed between performance immediately post-training and six months post-training in the theoretical questionnaire (p<0.001, PCT: moderate), CPR OSCE (p<0.001, PCT: moderate), and FBAO OSCE (p<0.001, PCT: weak).

Regarding the satisfaction form, Cronbach’s alpha was 0.817, indicating good internal consistency of responses (Table 4). Correlating student performance with satisfaction responses indicated that higher self-reported confidence in CPR (p<0.001) and FBAO scenarios (p<0.05) was associated with an increased likelihood of recommending the program.

**Table 4:** Satisfaction scores based on responses to a Likert scale questionnaire

Question presented using the Likert scale	Average ranking	If item is deleted Cronbach’s α
Chance of program recommendation	4.82	0.809
Perception of the program’s organization	4.60	0.805
Perception of self-confidence to act in CPR situations	3.94	0.798
Perception of self-confidence to act in FBAO situations	4.11	0.798
Perception of the theoretical questionnaire’s difficulty	3.52	0.819
Perception of feedback received after practice	4.48	0.788
Perception of learning through practice stations	4.65	0.796
Perception of evolution between practices before and after training	4.57	0.797
Perception of program duration	4.49	0.793
Degree of satisfaction with the program	4.66	0.796

CPR: cardiopulmonary resuscitation; FBAO: foreign body airway obstruction.

## DISCUSSION

The intervention implemented within the study resulted in a statistically significant improvement in students’ theoretical knowledge of CPR and FBAO scenarios. Practical performance across all competencies assessed during the OSCE also showed marked enhancement. The results further demonstrated the progression of knowledge and performance before and after training, with satisfactory retention of acquired competencies observed six months post-intervention.

The existing literature presents divergent views on the effect of age on students’ performance in first-aid training.^(18-20)^ In the current study, no differences were noted in students’ knowledge or performance before and immediately after training. Nonetheless, younger students demonstrated superior retention of theoretical knowledge six months post-training. As previous research has included participants from different age groups, direct comparison with the current results was restricted.

Earlier studies by Cömert et al. and Jones at al. reported that boys perform deeper chest compressions than girls during CPR.^(15,20)^ In contrast, the current study found that female students outperformed their male counterparts in the subsequent CPR OSCE. The cause of this difference is unclear, as variables such as weight, height, and body mass index—previously associated with CPR performance—were not included in this study’s measurements.^(15,20)^

Prospective analysis revealed no significant differences in pre- or post-training knowledge and performance among students across first, second, and third years. Likewise, students with a history of academic failure did not display learning deficits. These outcomes are consistent with findings from Mohd Sharif et al., who observed no significant differences in first-aid knowledge among cohorts in their study spanning 30 schools.^(21)^

The first question of the TQ concerning the correct emergency number in Pernambuco (192) achieved the highest correct response rate post-training. This is particularly significant, as the interval between an emergency’s occurrence and the arrival of the Mobile Emergency Care Service (SAMU - *Serviço de Atendimento Móvel de Urgência*) is critical for patient survival.^(17)^ Six months after training, the correct response rate decreased to 88.9%, but remained higher than the pre-training rate.

Pre-training responses to three TQ questions covering key CPR parameters and one question about AED use had correct rates below 15%, indicating unfamiliarity and misconceptions about appropriate techniques. Statistically, students with no prior exposure would be expected to select the correct answer 25% of the time. These four questions experienced the greatest increase in correct responses immediately after training, yet also saw the sharpest decline after six months. However, accuracy rates remained statistically above pre-training levels, likely reflecting the specificity of the subject matter and the tendency for knowledge to decay without regular practice. This pattern is commonly observed in specialized topics, where long-term retention is closely linked to regular practice and the continuous application of acquired knowledge.

Questions related to choking scenarios showed the smallest gains in accuracy immediately after training, but were the only ones for which the correct response rates increased after six months.

Post-training TQ scores indicated satisfactory overall student performance. Nevertheless, although accuracy more than doubled, only approximately half of the students demonstrated adequate competence in questions on the correct compression site, effectiveness parameters, and the sequence of actions during emergencies.

Significant improvements in practical performance were observed in OSCE scores from pre- to post-training. Before training, the median CPR OSCE score was 0.00, reflecting limited exposure to such scenarios. Post-training, the median score increased substantially, yet remained between 40% and 60% of the maximum possible score. Six months later, performance declined further, typically falling within the 35-50% range.

Cumulatively, these results confirmed the utility of the OSCE as a tool for evaluating student proficiency in CPR and FBAO, with a moderate correlation between post-training TQ scores and OSCE performance for both competencies. This underscores the value of the OSCE in assessing student outcomes following first-aid training.

A study conducted in pediatric environments in Japan found that immediate CPR and access to an AED increase survival chances in schools by approximately fourfold.^(22)^ Similarly, a multicenter study by Kua et al. involving 1,196 students aged 11-17 years demonstrated an increase in the willingness to use an AED from 11.7% pre-training to 78.0% post-training.^(23)^ These findings reinforce the importance of integrating CPR and AED training programs into school curricula.

Comparison of post-training OSCE scores for CPR—with and without AED-related parameters—showed a higher percentage increase in the median score when AED parameters were included (66.7% *versus* 56.2%), based on respective maximum scores (6.00 and 8.00, respectively). This is particularly relevant given the limited public access to AEDs in Brazil^(24)^ and suggests that OSCE-based training promotes consistent student performance in real-world cardiac arrest scenarios.

This study has certain limitations. First, neither the TQ nor OSCE parameters underwent expert validation or reliability calculations; rather, their development was based solely on the guidelines provided by the American Heart Association. This may affect the reliability and content validity of the assessment tools. Furthermore, the disproportionate distribution of topics within the TQ could influence analytical outcomes, particularly the observed correlation between OSCE performance for FBAO and questionnaire results. Second, the study design lacked randomization between participating schools and a control group, thereby compromising internal validity and limiting causal inference. The use of handcrafted manikins, while practical and cost-effective for large-scale assessment, limits the accuracy of CPR performance measurement and may compromise evaluation precision. Third, the study employed a convenience sampling method, which inherently constrains the generalizability of the results to a broader student population. A substantial attrition rate was observed in the final phase of the study, potentially compromising the robustness and statistical power of the dataset. This loss of participants may have been influenced by a range of external variables, including academic demands and logistical challenges faced by the students.

Future research should prioritize expert validation of assessment instruments, strive for a more balanced representation of theoretical content, and adopt robust strategies aimed at minimizing participant attrition and enhancing sample representativeness.

## CONCLUSION

Simulation-based first-aid training scenarios exhibit positive, albeit moderate, effects on improving knowledge and performance among high school students, regardless of age, grade, parental education, or history of academic failure. The application of handcrafted manikins in the objective structured clinical examination has been validated as an effective and economical resource for student learning and assessment, making it particularly suitable for educational settings with limited resources. Given its feasibility and potential benefits, integrating the objective structured clinical examination approach with affordable simulation models should be considered a strategy to strengthen first-aid education in schools. Nonetheless, additional studies are required to assess long-term knowledge retention and to optimize the implementation of objective structured clinical examination -based training programs in secondary education curricula.
